# Assessing the Influence of Confining Pressure on the Consolidation of Granular Bulk Models Using an Integrated Sensor System

**DOI:** 10.3390/s26010277

**Published:** 2026-01-01

**Authors:** Evgenii Kozhevnikov, Mikhail Turbakov, Zakhar Ivanov, Daniil Katunin, Evgenii Riabokon, Evgenii Gladkikh, Mikhail Guzev

**Affiliations:** Laboratory of Natural Gas Hydrates, Perm National Research Polytechnic University, Perm 614990, Russia; msturbakov@pstu.ru (M.T.); zakharivanov@pstu.ru (Z.I.); dekatunin@pstu.ru (D.K.); riabokon@pstu.ru (E.R.); gladkih.ea@pstu.ru (E.G.); guzev@iam.dvo.ru (M.G.)

**Keywords:** confining pressure, porosity, permeability, granular bulk model

## Abstract

Large-scale bulk models offer a promising approach for the experimental investigation of flow in porous media. However, conventional configurations frequently lack adequate confinement systems, resulting in model instability under dynamic flow conditions. This paper introduces a novel experimental apparatus designed for large-scale porous media flooding studies. The porous medium is represented by a tubular granular bulk model measuring one meter in length and 95 mm in diameter. An integrated array of distributed pressure, temperature, and electrical resistance sensors allows for the acquisition of a longitudinal pressure profile, the evaluation of the model’s consolidation state, and the assessment of its stress sensitivity. Comparative studies of filtration processes are presented for a granular bulk model under both confined and unconfined conditions. The results indicate that in the absence of confinement, the model exhibits high sensitivity to pressure differentials, manifesting as a nonlinear relationship between flow rate and pressure drop alongside significant fluctuations in electrical resistance. Conversely, cyclic loading under confining pressure promotes uniform and stable consolidation of the model, thereby minimizing hysteresis and particle displacement. These findings underscore that effective confinement is critical for ensuring the representativeness of data derived from large-scale bulk models of unconsolidated porous media.

## 1. Introduction

The study of filtration laws in various media is carried out using experimental studies on samples of porous media [1]. Depending on the scale of investigation, experimental studies are divided into: micro-nano scale [2,3], which utilizes nano- and microfluidics; macro scale, involving core samples of rocks [4]; large-scale, typically using large granular bulk models [5]; and full-scale studies conducted on real porous media using geotechnical or production wells [6]. Micro- and nanofluidics are illustrative, but transferring their research results to a real scale is difficult [7]. Macroscale studies using core samples, despite the advantage of employing real rocks, are hampered primarily by material scarcity, frequent non-representativeness, and the inability to study the influence of heterogeneities [8]. The main disadvantage of large-scale studies is their frequent reliance on granular bulk models consisting of unconsolidated materials, most often quartz sand [9]. The use of glued sand models [10] complicates their fabrication and reuse. Full-scale studies are typically the most expensive and least controlled. Among all the modeling scales considered, large-scale models are the most promising in terms of controlling the media structure and the ease of transferring experimental data to full scale. Large-scale setups can also be used to study gravity flow separation [10]. Recently, a steady trend of increasing interest in experimental seepage studies using large-scale granular bulk models has been noted in the literature.

In geotechnical engineering, various soil columns [5,11] and sand tanks [12,13] are used to study seepage and solute transport. Such models often have a simple design in the form of vertical or horizontal pipes [14]. Electrical resistance sensors are placed inside the soil to assess the movement and dispersion of a salt solution front [11]. Ports are also placed on the walls for communication with the pipe interior and for pressure measurement. Wells can be installed throughout the model’s sand tanks to monitor pressure distribution. The transparent walls of soil tanks allow for the visual assessment of fluid migration [15].

Large-scale models [16,17] are used for research in high-viscosity oil production. In [17], a setup with a granular bulk model measuring 17.1 cm in diameter and 47 cm in length is presented; pressure ports are provided along the model’s length to estimate its permeability during injection. In [16,17], the models are located in a vertical pipe, and sand consolidation is achieved by axial loading. In [17], the maximum consolidating pressure is 690 kPa. In [16], in addition to axial loading, a confinement system is present that allows for a pressure of 2 MPa to be applied. In these setups, filtration is performed from top to bottom; this scheme carries a high risk of gravity influencing the combined flow of oil and water. Uneven consolidation of the granular bulk model along its height is also possible due to the weight of the sand itself. Methods for controlling the degree of consolidation are also lacking. The example in [10] demonstrates that consolidation of granular bulk models can be achieved by gluing sand grains together with epoxy resin. Furthermore, the setup presented in [10] features elevated pressures—up to 70 MPa—and a distributed network of acoustic-electric scanning sensors, allowing for the assessment of displacement front progression. In [18], a pseudo-triaxial cell with a granular bulk model was used to study the effect of deviatoric stress on the porosity and permeability of granular bulk models at a maximum confining pressure of 1.2 MPa. However, this model lacks sensors for determining filtration parameters along its length.

Large-scale granular bulk models are widely used in studies of natural gas hydrate production technologies. A visual simulator for assessing gas hydrate formation is presented in [19]. In this study, the granular bulk model is housed in a 1-m-long, 2.54-cm-diameter reactor. The advantage of this setup is the ability to visually assess gas hydrate formation at a pressure of 9.5 MPa. Electrical resistivity sensors are uniformly distributed along the reactor, allowing for the assessment of hydrate formation sites. Reference [20] presents an experimental apparatus in which a granular bulk model is housed in a high-pressure reactor capable of withstanding a pressure of 15 MPa. This setup allows for the visualization of hydrate formation using magnetic resonance imaging. The granular bulk model is consolidated by dense backfill without applying confining pressure. Reference [9] presents a fan-shaped simulator. This simulator is a pizza-like segment with a radius of 3 m, capable of withstanding a pressure of 15 MPa. This setup is designed to study temperature evolution around a well in hydrate-bearing sediments during depressurization. It is intended for studying granular bulk models of bottom sediments without consolidation, as it lacks confining pressure. Thus, based on an analysis of the existing literature, it has been established that the effect of confining pressure on sand consolidation has not been adequately studied. Based on the identified shortcomings in the literature, this study addresses two primary gaps: the lack of established methods for implementing controlled cyclic loading in large-scale granular models, and the insufficient understanding of how such cyclic loading and the resulting strain heterogeneity influence model consolidation. The objectives of this article are therefore: (1) To present the design of a novel large-scale porous media flooding unit capable of applying controlled confining pressure and cyclic loading. (2) To experimentally investigate the effects of confining pressure and cyclic loading on the consolidation behavior, stability, and filtration properties of a granular bulk model through a comparative study of tests conducted with and without confinement.

## 2. Materials and Methods

**Figure 1:**
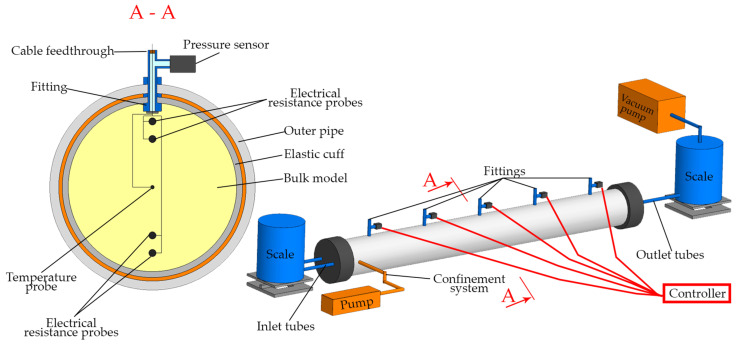
Schematic representation of the experimental setup for large-scale flooding.

The large-scale porous media flooding unit consists of a porous model formed by sand packed within an elastic cuff housed inside a pipe (Figure 1). The annulus between the cuff and the pipe wall serves as a space for fluid supply and the application of confining pressure. The pipe ends are sealed with specialized caps, which perform three functions: sealing the elastic cuff, isolating the annular confinement space, and providing ports for fluid injection and drainage from the model. The self-made three-piece design of these end caps (Figure 1) enables the model to be filled with sand without requiring disassembly of the entire unit.

Five sensor arrays, herein referred to as “bundles,” are positioned at equidistant 20 cm intervals along the length of the granular bulk model. Each bundle comprises one pressure sensor (Sendo Sensor, Ningbo, China), one temperature probe (Eaton, OH, USA), and two pairs of electrical resistance probes. All sensors are connected via wiring to a single programmable logic controller (Arduino, Ivrea, Italy), which records data synchronously.

The temperature and electrical resistance probes are situated within the bulk model, as illustrated in Figure 1, section A-A. The temperature probes are centrally located to monitor temperature and its evolution during the flow of liquids at different temperatures. The two pairs of electrical resistance probes are arranged to measure the electrical resistance of the porous medium in the upper and lower sections of the model. This configuration enables the assessment of potential gravitational flow stratification and the degree of sand consolidation when the model is oriented horizontally. Electrical resistance probes serve as a valuable diagnostic tool for determining not only fluid saturation but also porosity [21,22].

The pressure sensors are externally mounted and connected to the interior of the bulk model via a fitting and a short 6 mm diameter tube. In a homogeneous porous medium, pore pressure is uniformly distributed across the model’s cross-section; therefore, this lateral placement allows for accurate fluid pressure measurement. The longitudinal positioning of pressure sensors along the model enables the acquisition of a transient pressure profile during fluid injection. The absolute measurement range of the pressure sensors is 50–300 kPa, and they were calibrated using a reference pressure gauge.

Fluid is supplied to the bulk model from an inlet tank placed on a scale and is discharged into a similar outlet tank also placed on a scale. In this study, flow is induced by applying a low vacuum (absolute pressure = 91.3 kPa) to the outlet tank. This method ensures a constant pressure differential across the model while simultaneously facilitating the straightforward measurement of fluid mass flow at both the inlet and outlet. Maintaining a constant pressure drop eliminates external pressure pulsations typically associated with pump operation. Furthermore, the low vacuum at the outlet provides additional effective stress, contributing to the compaction of the bulk model. The pressure in the discharge line is regulated by a vacuum regulator, and a 100-L receiver connected to the low-vacuum line dampens any residual pressure fluctuations. The temporal change in liquid mass within the tanks during pumping characterizes the flow rate, q, in mL/s.

**Figure 2:**
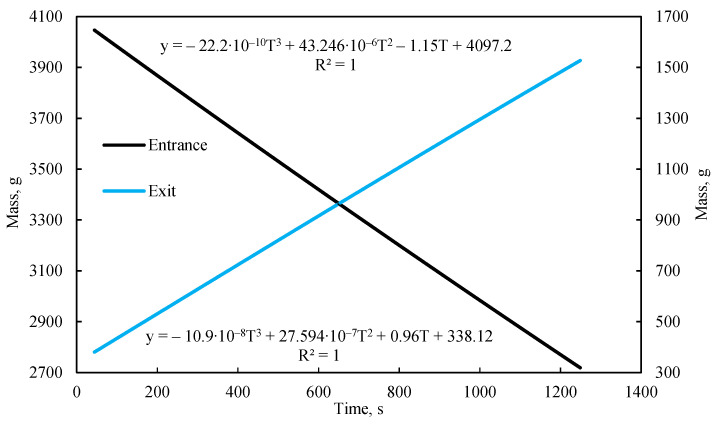
The change in water mass within the containers and an example of the flow function approximation over time.

A characteristic of this gravimetric flow measurement method is that the hydraulic head in the inlet tank decreases as it empties, resulting in a gradual reduction in the overall pressure differential across the model. Consequently, the liquid flow rate is nonlinear over time. However, this nonlinearity is very smooth and scarcely noticeable on the mass-change graph (Figure 2), allowing the flow regime to be considered quasi-steady-state. Given this gradual decline, the hydraulic head can be treated as constant over short intervals (e.g., one second), equal to its average value during that interval. Thus, the instantaneous fluid flow rate can be represented by an approximating function fitted to the actual change in fluid mass. In this work, the mass change during quasi-steady-state flow is best approximated by a cubic function of time, an example of which is shown in Figure 2.

The bulk model is composed of quartz sand with defined particle size and morphology, contained within an elastic sleeve. To prevent suffusion, graded gravel filters with progressively coarser particles are installed at both ends of the model. The experimental configuration facilitates the construction of porous media models with targeted porosity and permeability by selecting specific fractions of sand, clays, and other granular materials. It enables the creation of reservoir models with diverse architectures, including both layered and homogeneous structures, with the specific design contingent upon the research objectives.

Consolidation of the bulk model to inhibit sand movement is achieved through dense packing combined with confining pressure applied to the elastic sleeve. This dense packing is attained by evacuating the annular space between the sleeve and the outer casing pipe during the filling process. The elastic sleeve, which has an initial diameter smaller than the pipe’s inner diameter, expands to accommodate additional sand. This filling method mitigates the effects of sand shrinkage on subsequent model deformation and ensures maximum initial consolidation. The sand was introduced via a narrow hose, a technique that eliminated a free-fall phase for the grains, thereby preventing gravitational segregation. As a result, a high degree of homogeneity was achieved despite the use of polydisperse sand. Confining pressure is subsequently generated by pumping water into the annular space between the elastic sleeve and the outer casing pipe.

Advantages of the present setup relative to comparable models include:

1. Larger dimensions (95 mm diameter, 1000 mm length). The increased cross-sectional diameter enables assessment of the displacement front progression through the formation thickness, providing refined data on factors influencing the front profile. The larger scale also reduces the relative impact of measurement errors at low fluid velocities, facilitating more accurate simulation of flow in distant reservoir zones.
2. A confinement system that enables porous medium consolidation and permits study of the effect of confining pressure on porosity, permeability, and electrical resistance.
3. An integrated sensor array for monitoring pressure, temperature, and electrical resistance along the entire model length.
4. Flow rate control via outlet pressure regulation, which ensures a stable pressure differential across the model and maintains a steady flow regime.
5. A horizontal orientation and large scale that allow for the physical modeling of gravitational effects and reservoir heterogeneities on filtration processes and flow profiles.

**Figure 3:**
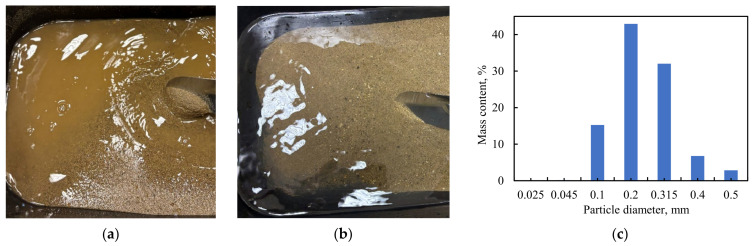
Sand preparation (**a**) before washing, (**b**) after washing, (**c**) fractional composition of sand used in this study.

For this study, to ensure precise control of experimental parameters, the sand was washed (Figure 3a) until the effluent water was clear (Figure 3b). The washed sand was subsequently dried and sieved through a 0.5 mm mesh. A sieve analysis of the prepared sand is presented in Figure 3c. The median particle diameter (d_50_) was 0.24 mm. Post-washing, the content of sand grains smaller than 0.1 mm in diameter was 0.16%.

**Figure 4:**
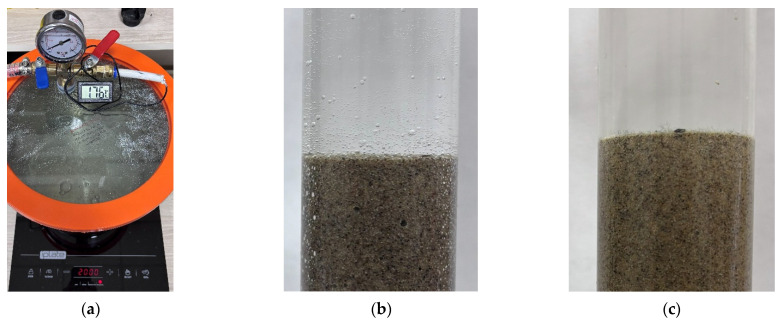
Vacuum degassing and heating of water for flooding (**a**). Release of dissolved gas in free volume and pore space when using untreated water (**b**). Absence of gas bubbles when using vacuum-degassed water (**c**).

Vacuum-degassed tap water was used for both saturation and flooding (Figure 4a). The water was evacuated and heated to 25 °C to remove dissolved gases (Figure 4b). Experimental studies indicate that vacuum degassing at a pressure of approximately 6 kPa is sufficient to prevent the release of dissolved gases within the bulk model and to maintain constant saturation (Figure 4c), provided the absolute pressure does not fall below 60 kPa. At lower absolute pressures, water begins to boil within the porous medium. In this study, the absolute pressure in the bulk model was maintained above 80 kPa. The algaecide monolinuron was added to the water to prevent algal growth and the consequent clogging of the porous medium. An algicide prevents plant photosynthesis and excessive algae growth; the choice of algicide type is based on its relative safety for living organisms and laboratory personnel.

The presence of a confinement system and the uniform distribution of pressure, temperature, and electrical resistance sensors along the length of the setup are its key distinctive features. This configuration enables a comprehensive assessment of the influence of confining pressure on unconsolidated porous media and their filtration properties—the primary objective of this study. The research methodology involves a comparative analysis of filtration parameters, including pressure drop, flow rate, and electrical resistance, across various flow regimes under both confined and unconfined conditions. Additionally, the effect of confining pressure on pore volume was investigated by incrementally adjusting the confining pressure while simultaneously measuring the corresponding water volume expelled from the saturated bulk model. All experiments were conducted with the model in a horizontal orientation. The results of these comparative tests and their discussion are presented in the following section.

## 3. Results and Discussion

### 3.1. Bulk Model Without Confinement

**Figure 5:**
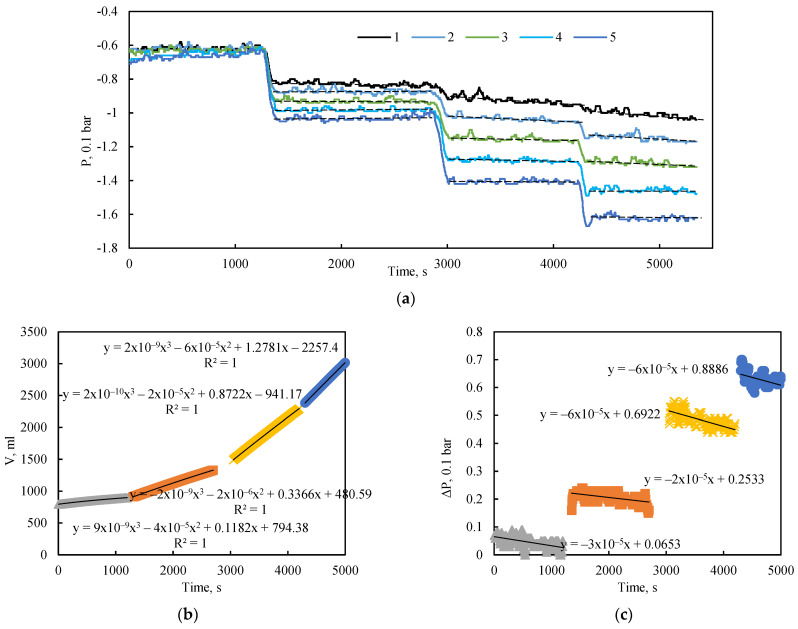
(**a**) Dynamics of pressure at the sensor locations. Dashed black lines indicate the average pressure for each quasi-steady-state flow regime; solid black lines represent the corresponding approximations. (**b**) Change in the cumulative volume of injected fluid in the outlet tank over time. (**c**) Pressure drop across the entire length of the bulk model as a function of injection time at 0 kPa confining pressure.

The placement of pressure sensors enables the acquisition of a longitudinal pressure profile within the bulk model during fluid flow. Figure 5a illustrates the pressure dynamics along the model over time during flooding experiments with a stepwise increase in the inlet-outlet pressure differential from 5 to 20 kPa, under a confining pressure of 0 bar (0 kPa).

These unconfined flooding tests revealed that a quasi-steady-state flow regime, characterized by constant pressure readings at all measurement points, was quickly established following each change in extraction pressure (Figure 5a). The figure also shows a slight negative slope in the average pressure trend over time, attributable to the declining hydraulic head in the inlet supply tank. This effect is most pronounced in the initial section of the model, near sensors 1 and 2. The slope of this pressure decay is also dependent on the applied pressure drop; a higher differential pressure induces a greater flow velocity and, consequently, a more rapid decrease in the hydraulic head.

The corresponding flow rate for each flow regime was determined using cubic equations fitted to the change in liquid mass within the outlet tank over time (Figure 5b). The total pressure drop across the length of the model, defined as the difference between the first and fifth pressure sensors, is represented by the approximation of pressure change over time shown in Figure 5c.

Data analysis also identified low-frequency pressure fluctuations, even in the absence of external perturbations. These fluctuations are not attributable to measurement error, as their amplitude significantly exceeds the sensor’s error margin. They are most likely associated with internal flow stabilization processes. The results confirm that the sensitivity of the pressure sensors is sufficient for accurate pressure measurement during fluid flow.

**Figure 6:**
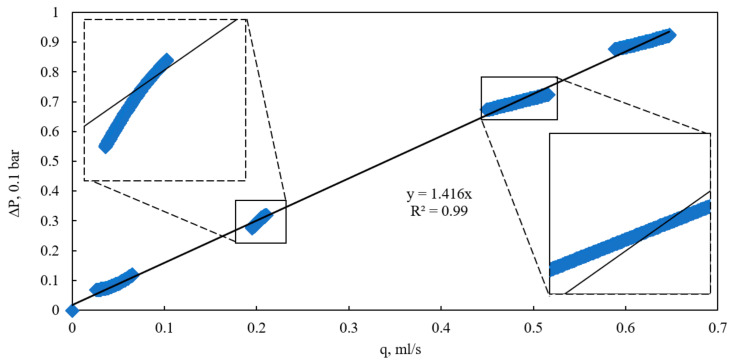
Dependence of pressure drop on flow rate at various quasi-steady flow conditions and confining pressure of 0 kPa.

Based on the calculated values, a plot of the pressure drop across the full length of the bulk model versus the flow rate was constructed for various quasi-steady-state flow regimes under a confining pressure of 0 kPa (Figure 6). The approximation of the flow rate–pressure drop data exhibits a classical linear form with an intercept near the origin, characterizing the flow within this range as Darcy flow. This conclusion is further supported by the calculated Reynolds number, which did not exceed 0.08 even at the highest flow rates, confirming the purely viscous, laminar regime. In Darcy flow, the pressure drop results from viscous friction between the fluid and the pore walls during laminar motion, with deviations attributable to additional physical factors [23].

However, despite the low Reynolds number, it was observed that at flow rates exceeding 0.2 mL/s, the slope of the experimental flow rate–pressure drop curves diverges from the general linear Darcy approximation. At lower flow rates (below 0.1 mL/s), the data align closely with the theoretical Darcy relationship. The variation in slope among different quasi-steady-state regimes is most likely attributable to the influence of the internal flow-induced pressure drop on the consolidation state of the bulk model under conditions of zero external confining pressure and the absence of uniform compression. Pressure changes within these regimes can significantly affect local porosity, potentially inducing grain displacement, compaction, or dilation [24].

**Figure 7:**
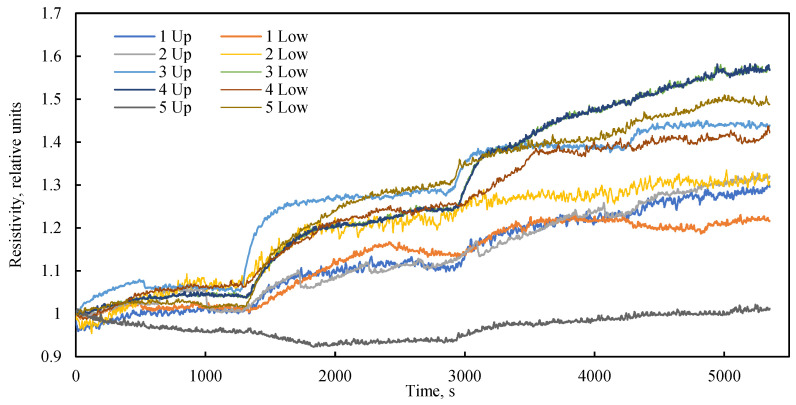
Dynamics of relative electrical resistance along the length of a bulk model at different liquid flow rates over time at a confining pressure of 0 kPa.

The experimental setup incorporates electrical resistivity probes as a supplementary diagnostic tool for assessing alterations in the porous structure during filtration or in response to external stresses. It is established that in porous media, zones of higher porosity typically exhibit lower electrical resistivity compared to zones of lower porosity. Figure 7 illustrates the temporal dynamics of relative electrical resistivity along the length of the bulk model under various flow regimes and a confining pressure of 0 kPa. Throughout the experiment, a steady increase in the bulk model’s electrical resistivity was observed. Stepwise changes in resistivity coincided with transitions between flow regimes. The pattern of resistivity change was found to be consistent across nearly all probes, confirming a uniform, bulk tendency for volumetric change within the model during fluid flow in the absence of confining pressure.

**Figure 8:**
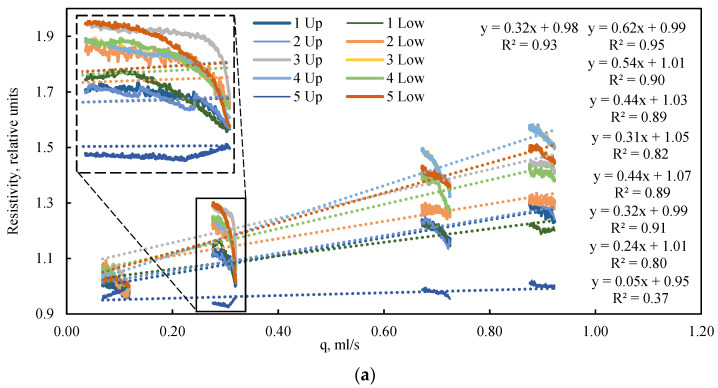

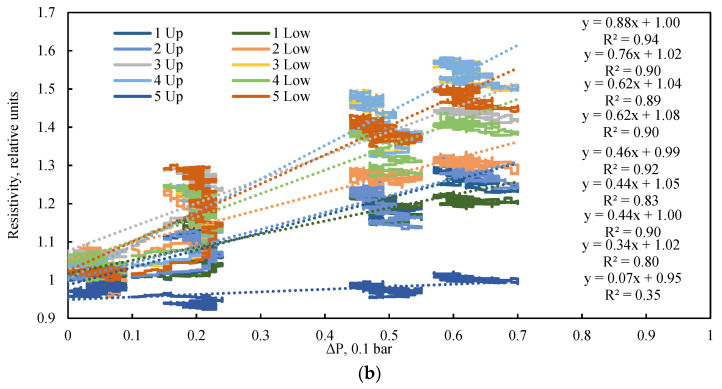
Dependence of relative electrical resistance on (**a**) flow rate and (**b**) pressure drop under various quasi-steady-state flow conditions and a confining pressure of 0 kPa. The highlighted region illustrates the inverse relationship between electrical resistance and flow rate during quasi-steady-state operation. Dotted lines are approximations.

An analysis of the electrical resistance dynamics reveals a clear dependency on the fluid flow rate (Figure 8a), or more precisely, on the driving pressure differential (Figure 8b) that induces this flow. In the absence of confining pressure, the low vacuum applied to the model outlet results in the compression of the elastic sleeve by atmospheric pressure. A lower absolute pressure (i.e., a stronger vacuum) leads to greater compression of the model and a corresponding increase in its electrical resistance. Consequently, despite the initial dense packing, the bulk model exhibits high sensitivity to the pressure drop generated by the fluid flow itself. This phenomenon is frequently overlooked in studies of bulk models operating without external confinement. The data on electrical resistance obtained under cyclic loading can be utilized for the numerical simulation of porosity changes within the bulk model during confinement, employing the established linear dependence between electrical resistance and porosity.

### 3.2. Effect of Confining Pressure on Porosity

**Figure 9:**
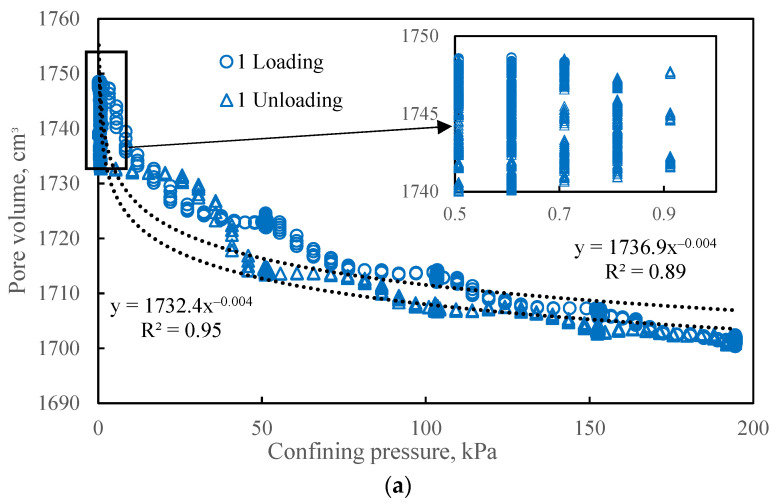

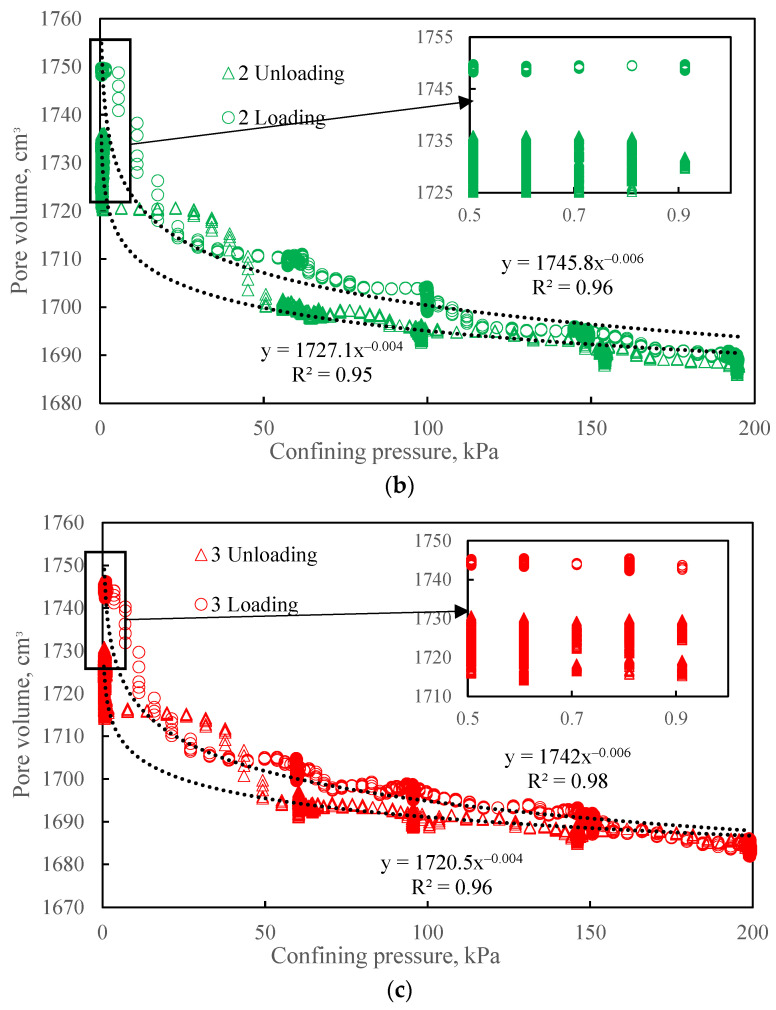
The effect of confining pressure on the pore volume of a bulk model after 1 (**a**), 2 (**b**), and 3 (**c**) loading cycles. Circles represent the loading path; triangles represent the unloading path.

The effect of confining pressure on porosity was investigated by measuring the mass of water expelled from the saturated bulk model in response to a stepwise increase in confining pressure from 0 to 190 kPa, followed by unloading. Three complete loading and unloading cycles were performed. The results, presented in Figure 9, show the change in the pore volume of the bulk model as a function of confining pressure.

The change in pore volume is best described by a power-law relationship (R^2^ from 0.89). Hysteresis in pore volume was also observed: during the unloading phase, the pore volume curve lies below the corresponding loading curve. However, despite this intermediate hysteresis, the pore volume fully recovered its initial value upon complete removal of the confining pressure in all cycles. The hysteresis during unloading is attributed to inter-granular friction, which impedes the recovery of the initial grain packing structure [25]. The complete restoration of pore volume in the absence of confining pressure is likely enabled by the elasticity of the model’s containing cuff. Consequently, for studies focusing on the effect of confining pressure on the porosity and permeability of unconsolidated granular media, it is not recommended to reduce the confining pressure to zero, as this may mask inelastic compaction effects.

The pattern of pore volume change under cyclic loading is consistent across all cycles. However, determining the stress sensitivity of bulk models solely through volumetric methods provides an incomplete understanding of the internal processes [26], and cannot assess potential deformation inhomogeneities. The availability of supplementary tools to evaluate the degree and uniformity of consolidation is therefore critical for validating the representativeness of bulk models relative to actual consolidated formations. Furthermore, graphs of porosity versus confining pressure do not indicate the minimum pressure required to achieve a consolidated state, nor the number of loading/unloading cycles sufficient to equalize the compaction of unconsolidated sand throughout the model’s dimensions. Assessing compaction uniformity is only feasible using internal monitoring of the porous medium.

**Figure 10:**
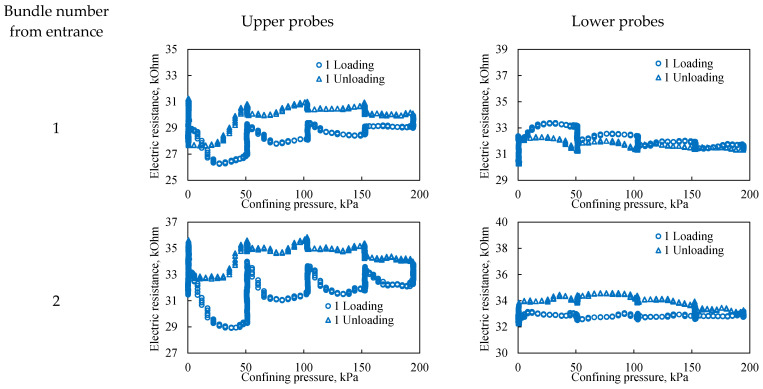

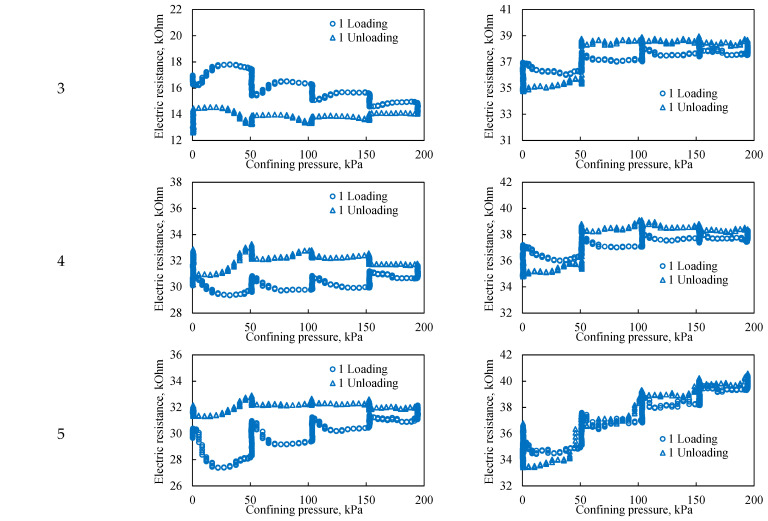
The effect of confining pressure on the electrical resistivity of a bulk model under one loading cycle. The graphs on the left represent probes located in the upper part of the bulk model, while the graphs on the right represent probes located in the lower part of the bulk model. The number from entrance corresponds to the sensor bundle number: 1 is located at the beginning of the bulk model, and 5 is located at the end of the bulk model. The distance between sensor bundles is 20 cm.

**Figure 11:**
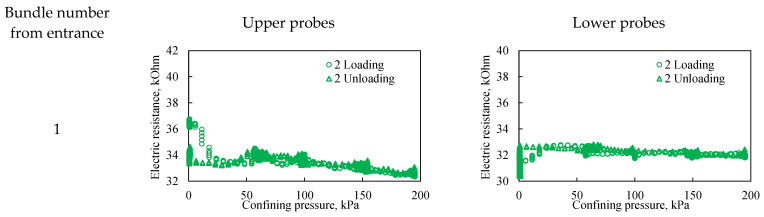

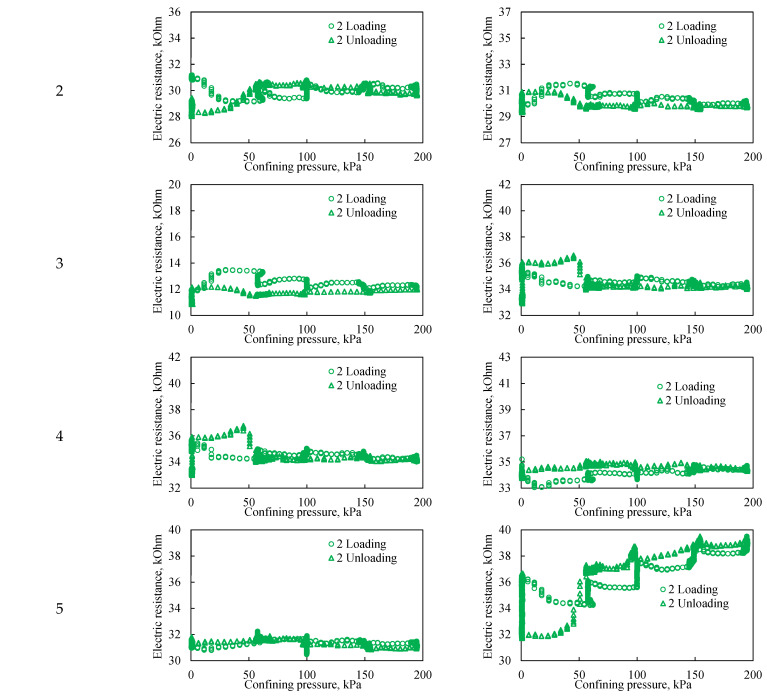
The effect of confining pressure on the electrical resistivity of a bulk model under two loading cycles. The graphs on the left represent probes located in the upper part of the bulk model, while the graphs on the right represent probes located in the lower part of the bulk model. The Number from entrance corresponds to the sensor bundle number: 1 is located at the beginning of the bulk model, and 5 is located at the end of the bulk model. The distance between sensor bundles is 20 cm.

**Figure 12:**
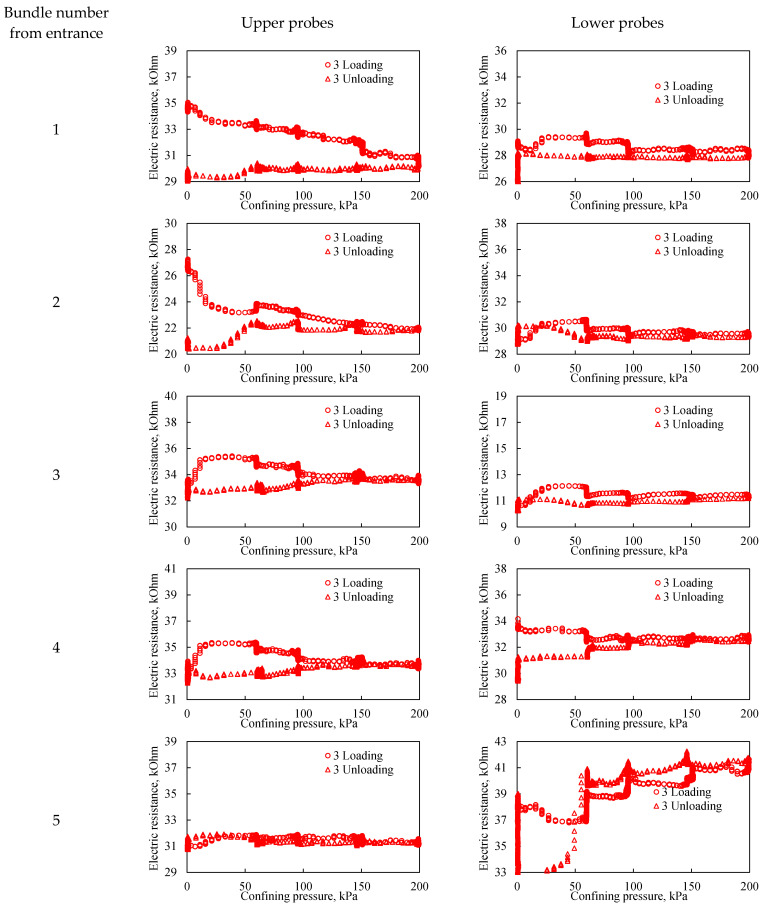
The effect of confining pressure on the electrical resistivity of a bulk model under three loading cycles. The graphs on the left represent probes located in the upper part of the bulk model, while the graphs on the right represent probes located in the lower part of the bulk model. The number from entrance corresponds to the sensor bundle number: 1 is located at the beginning of the bulk model, and 5 is located at the end of the bulk model. The distance between sensor bundles is 20 cm.

In this work—a distinguishing feature from other studies—this internal monitoring is achieved through electrical resistance testing, which provides a non-destructive assessment of the *in situ* state of the porous medium under varying confining pressure. Representative resistivity curves are presented in Figure 10, Figure 11 and Figure 12. In these figures, the graphs on the left correspond to probes located in the upper part of the bulk model, while those on the right correspond to probes in the lower part. The probe number indicates its position relative to the fluid inlet, with bundle 1 at the model inlet and bundle 5 at the outlet, spaced at 20 cm intervals.

Figure 10 shows that during the first loading cycle, the dependences of electrical resistivity on confining pressure in the upper and lower parts of the bulk model differ significantly. Data from the upper probes indicate that the upper part of the model is initially less consolidated. This leads to significant hysteresis in electrical resistivity during loading and unloading, which directly reflects the more intense restructuring of the pore space in this zone. All probes, with the exception of the upper probes of the third bundle, record higher resistivity during unloading compared to loading. This phenomenon is explained by additional consolidation of the sand during the unloading phase, which is associated with stress redistribution within the model and reorientation of sand grains. Since electrical resistivity is sensitive to pore geometry and contacts between grains, its increase indicates irreversible compaction of the structure and a decrease in the conductivity of current paths. Probes in the lower part exhibit smaller hysteresis in electrical resistivity. This is directly related to the initial gravitational compaction of the lower layers of the model under the effect of its own weight. A higher initial density means that the pore structure here is less susceptible to further significant changes under confining pressure, which is reflected in more stable and reproducible resistivity readings. Thus, the different responses of the upper and lower probes directly illustrate the influence of gravity on the initial porosity distribution and the subsequent mechanical behavior of the unconsolidated granular medium.

During the second loading cycle, electrical resistivity hysteresis is significantly less pronounced across all probes compared to the first cycle (Figure 11). This indicates a more uniform deformation of the bulk model in both the longitudinal and vertical directions. Furthermore, within the observed pressure range, the dependence of electrical resistivity on confining pressure becomes negligible at pressures exceeding 60 kPa. At confining pressures below 60 kPa, however, a significant change in resistivity is observed, attributable to sand grain movement and associated changes in bulk model porosity.

The third loading cycle exhibits a pattern virtually identical to the second. The primary distinction is that in the third cycle, resistivity hysteresis is observed up to a higher confining pressure of approximately 100 kPa. This indicates the bulk model undergoes further significant compaction with successive cyclic loading. It is therefore established that cyclic loading can achieve a high degree of model consolidation, effectively bringing its geomechanical and hydraulic parameters closer to those of actual consolidated rocks.

Among the general characteristics, it is notable that electrical resistance hysteresis is most pronounced at the extremities of the bulk model, specifically within sensor bundles 1 and 5. Across all three cycles, the lower probes of bundle 5 (Figure 12) demonstrated a consistent pattern of electrical resistance changes. This consistency may not only reflect the local degree of consolidation under load but could also be influenced by potential artifacts introduced during the initial filling of the model. The stronger hysteresis at the ends of the model is caused by the presence of a gravel filter in close proximity to the sensor beams; this effect can be minimized by reducing the layers of the gravel filter.

### 3.3. Bulk Model Under Confining Pressure

**Figure 13:**
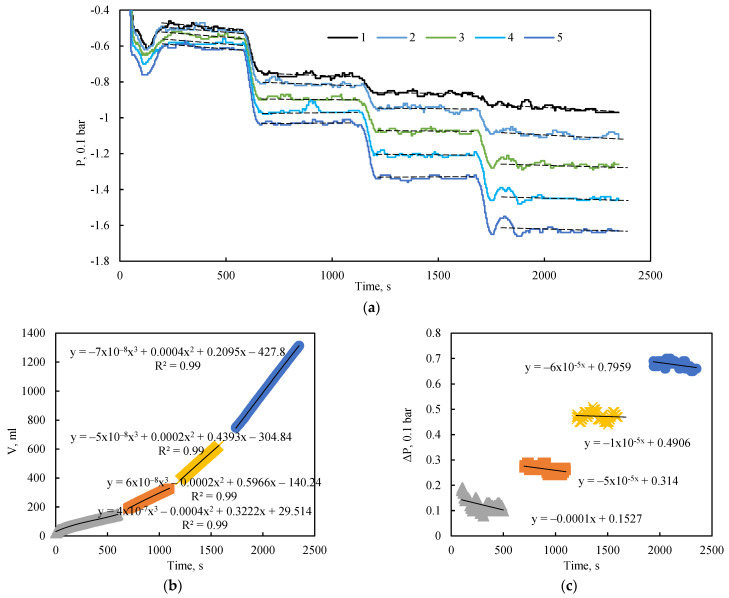
(**a**) Dynamics of pressure at the sensor locations. Dashed black lines indicate the average pressure for each quasi-steady-state flow regime; solid black lines represent the corresponding approximations. (**b**) Change in the cumulative volume of injected fluid in the outlet tank over time. (**c**) Pressure drop across the entire length of the bulk model as a function of injection time at 190 kPa confining pressure.

Waterflooding tests conducted under a confining pressure of 190 kPa demonstrated that a quasi-steady-state regime was rapidly established following each change in extraction pressure. This regime was characterized by constant pressure levels at all measurement points along the length of the bulk model (Figure 13a). Figure 13a also reveals a slight negative slope in the average pressure trend over time, which is less pronounced than in the unconfined tests due to the lower flow velocities in the consolidated model.

The flow rate for each regime was determined using cubic equations fitted to the temporal change in liquid mass within the outlet tank (Figure 13b). The total pressure drop across the entire length of the model, defined as the pressure difference between the first and fifth sensors, is represented by the approximation of pressure change over time shown in Figure 13c.

**Figure 14:**
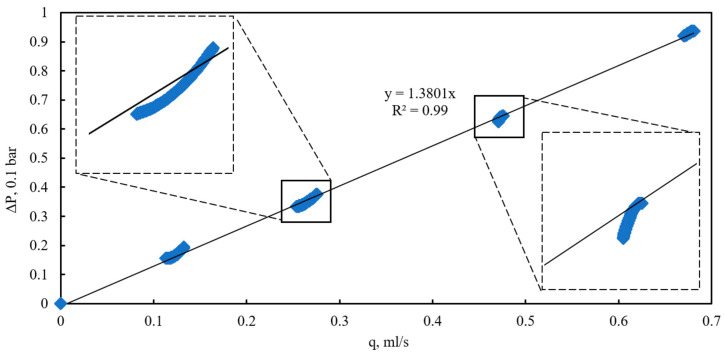
Dependence of pressure drop on flow rate at various quasi-steady flow conditions and confining pressure of 190 kPa.

Based on the calculated data, the pressure drop across the entire length of the bulk model is plotted against the flow rate for various quasi-steady-state flow regimes under a confining pressure of 190 kPa (Figure 14). The flow rate–pressure drop relationship exhibits a classic linear form with an intercept near zero, confirming Darcy flow under the given conditions. At a confining pressure of 190 kPa, the experimental curves align closely with the linear Darcy approximation, indicating the absence of significant extraneous factors influencing the flow. This alignment demonstrates that the bulk model is stable at this confining pressure, with minimal grain displacement occurring.

**Figure 15:**
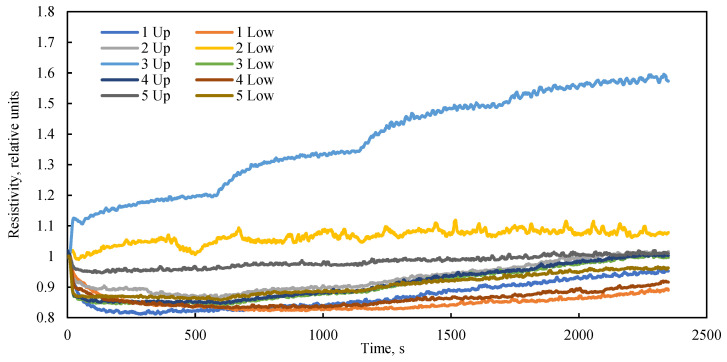
Dynamics of relative electrical resistance along the length of a bulk model at different liquid flow rates over time at a confining pressure of 190 kPa.

The dynamics of relative electrical resistance along the bulk model over time, under various flow regimes and a confining pressure of 190 kPa, are shown in Figure 15. Throughout the experiment, the electrical resistance of the bulk model remained virtually constant. The sole exception was the upper probe in the third sensor bundle, where minor variations may indicate localized artifacts within the model. The pattern of electrical resistance change was consistent across almost all other sensors, confirming the overall stability of the bulk model under a confining pressure of 190 kPa across the tested flow conditions.

**Figure 16:**
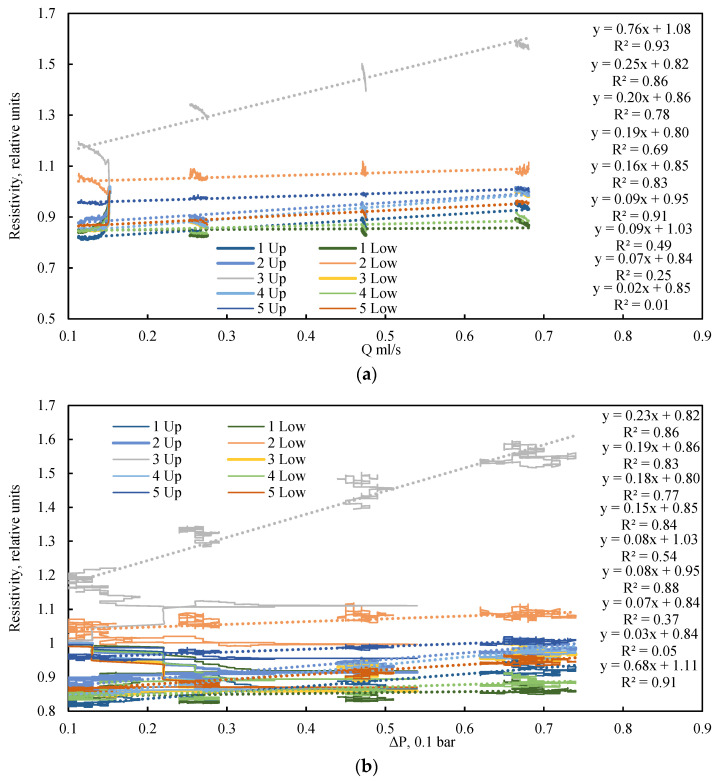
Dependence of relative electrical resistance on flow rate (**a**) and pressure drop (**b**) for various quasi-steady-state conditions and confining pressure of 190 kPa. Dotted lines are approximations.

An analysis of the electrical resistance dynamics reveals no clear dependency on either the fluid flow rate (Figure 16a) or the driving pressure drop (Figure 16b) when a confining pressure of 190 kPa is applied. This demonstrates that a confinement system is essential for ensuring the stability of bulk models. Furthermore, cyclic loading enhances model consolidation and extends the operable range for testing under varied fluid flow regimes.

## 4. Conclusions

This paper presents a new large-scale experimental setup and evaluates its capabilities for studying flow laws in porous media. The setup enables physical modeling of flow in large-scale porous media, which will ensure better transfer of laboratory test results to field scale. The developed method yields a highly consolidated model. Distributed pressure sensors and electrical resistivity probes enable the assessment of changes in the porous medium caused by both its deformation and the advancement of the displacement front.

The use of distributed electrical resistivity probes has proven effective for non-destructive testing of spatial compaction and model validation, which is critical for accurate modeling of flow processes.

The paired arrangement of electrical resistivity probes allows for the consideration of gravity flow separation in a volumetric model, as well as front advancement in a layered model.

Comparative flooding tests showed that in the absence of consolidating pressure, the porous volumetric model is very sensitive to pressure changes, a factor not always accounted for in such studies.

Cyclic loading promotes better consolidation of bulk models. With each loading and unloading cycle, the model exhibits less porosity hysteresis, but the model’s stabilization pressure, at which the sand grains are tightly packed, increases. As a guideline, it is recommended to determine the specific loading cycle at which pressure stabilization is achieved, characterized by minimal hysteresis in electrical resistance. In all subsequent experimental cycles, the confining pressure should not be reduced below this stabilization value.

Therefore, to ensure representativeness of the data obtained from large-scale volumetric models of unstable porous media, the use of a consolidation system is essential. To achieve uniform and stable model consolidation, cyclic loading for at least two cycles is recommended. Although this study utilized a specific porous medium, the fundamental deformation mechanisms and established physical relationships governing consolidation, hysteresis, and pressure sensitivity are universal for a variety of unconsolidated granular materials. Therefore, the key findings and methodological recommendations regarding volume confinement and cyclic loading are applicable to porous media with varying grain sizes or porosity.

## Data Availability

The data presented in this study are available on request from the corresponding author.
